# Endoscopic ear examination improves self-reported confidence in ear examination skills among undergraduate medical students compared with handheld otoscopy

**DOI:** 10.3205/zma001524

**Published:** 2022-02-15

**Authors:** Mohamed Bassiouni, Duha G. Ahmed, Samira Ira Zabaneh, Steffen Dommerich, Heidi Olze, Philipp Arens, Katharina Stölzel

**Affiliations:** 1 Charité – Universitätsmedizin Berlin, Campus Charité Mitte, Department of Otorhinolaryngology, Berlin, Germany

**Keywords:** otoscopy, otolaryngology, digital teaching, examination practical, ear pathology

## Abstract

**Objectives: **Handheld otoscopy is the standard tool used to teach ear examination in undergraduate and postgraduate medical education. Previous studies have shown that the undergraduate teaching of ear examination with handheld otoscopes is inadequate, resulting in low self-reported levels of student confidence in their diagnostic skills. With the increase in popularity of endoscopic ear surgery, an increasing number of otolaryngologists are using endoscopes for office examinations of the ear due to the method’s superior visualization and excellent image qualities. However, medical students usually do not receive exposure to endoscopic ear examination during their undergraduate curriculum. The aim of this study is to assess our preliminary experience with teaching endoscopic ear examination to undergraduate medical students.

**Methods: **A two-hour-long pilot practical course on basic ear examination was administered to undergraduate medical students with little to no previous experience with ear examination. The course was designed to minimize the duration of campus attendance and patient contact during the COVID-19 pandemic. The course included theoretical didactics, exemplary digital endoscopic images and peer physical practice of ear examination with both a handheld otoscope and a 0-degree endoscope. At the end of the course, the students completed a survey questionnaire consisting of eight questions mainly relating to their subjective confidence level with ear examination using either handheld otoscopes or endoscopes and their overall preference for either examination tool.

**Results:** Most students expressed a preference for ear examination with endoscopes over that with handheld otoscopes and reported an improved confidence level in their diagnostic ability with the former technique. The vast majority of students supported the teaching of endoscopic ear examination to future medical students.

**Conclusion:** The findings of this pilot project report and survey study support the early exposure of novice medical learners to endoscopic ear examination, which may help improve the confidence and diagnostic skill of medical students with regard to ear examination. The findings may have implications for the undergraduate teaching of ear examination in the post-COVID-19 era.

## Zusammenfassung

**Zielsetzung: **Die Otoskopie mit einem Hand-Otoskop ist das Standardverfahren in der Lehre von Ohruntersuchungen in der medizinischen Aus- und Weiterbildung. Frühere Studien haben gezeigt, dass der Unterricht mit Hand-Otoskopen in den ersten Semestern unzureichend ist, was dazu führt, dass die Studierenden nur wenig Selbstvertrauen in ihre diagnostischen Fähigkeiten entwickeln. Mit zunehmender Popularität der endoskopischen Ohrchirurgie verwenden immer mehr HNO-Ärzte Endoskope für Ohruntersuchungen in der Praxis, da diese Methode eine bessere Visualisierung und ausgezeichnete Bildqualität bietet. Allerdings werden Medizinstudenten während ihres Studiums in der Regel nicht mit der endoskopischen Untersuchung des Ohrs vertraut gemacht. Ziel dieser Studie ist es, unsere ersten Erfahrungen in der Lehre mit endoskopischen Ohruntersuchungen für Medizinstudenten zu bewerten.

**Methoden: **Wir haben einen zweistündigen praktischen Pilotkurs zur Basis-Ohruntersuchung für Medizinstudenten im Grundstudium durchgeführt, die bislang wenig oder gar keine Erfahrung mit Ohruntersuchungen hatten. Hierbei wurden die Dauer der Anwesenheit auf dem Campus und der Patientenkontakt während der COVID-19-Pandemie möglichst gering gehalten. Der Kurs umfasste theoretische Didaktik, digitale endoskopische Beispielbilder sowie gegenseitige praktische Untersuchungen des Ohrs (peer to peer) mit einem handgeführten Otoskop und einem 0-Grad-Endoskop. Am Ende des Kurses füllten die Studierenden einen Fragebogen mit acht Fragen aus, die sich hauptsächlich auf ihr subjektives Vertrauen in die Ohruntersuchung mit Hand-Otoskopen bzw. Endoskopen sowie auf ihre allgemeine Präferenz für eines der beiden Untersuchungsinstrumente bezogen.

**Ergebnisse: **Die meisten Studierenden zogen die Ohruntersuchung mit dem Endoskop gegenüber der Untersuchung mit dem Hand-Otoskop vor und gaben an, dass sie mit der endoskopischen Technik mehr Vertrauen in ihre diagnostischen Fähigkeiten hätten. Die überwiegende Mehrheit der Studierenden befürwortete die Vermittlung der endoskopischen Ohruntersuchung an künftige Medizinstudenten.

**Fazit: **Die Ergebnisse dieser Pilotprojektarbeit und der Umfrage untermauern die frühe Heranführung von Medizinstudenten an die endoskopische Ohruntersuchung. Dieses Verfahren kann dazu beitragen, das Selbstvertrauen und die diagnostischen Fähigkeiten der Medizinstudenten in Bezug auf die Ohruntersuchung zu verbessern. Die Ergebnisse könnten Auswirkungen auf die Lehre von Ohruntersuchungen im Grundstudium in der Zeit nach der COVID-19-Pandemie haben.

## 1. Introduction

The COVID-19 pandemic has posed great challenges in the delivery of medical education, yet, created vast opportunities for adjustments and innovations especially in digital undergraduate medical education, to reduce patient contact without compromising the learning experience. In the undergraduate otolaryngology curriculum at the Charité medical university, over 300 medical students are trained in ear examination every semester through bedside practical courses and patient examination modules. The delivery of this teaching format has proven especially challenging during the COVID-19 pandemic [1], since our university hospital experienced a high burden of COVID-19 patients as a regional COVID-19 treatment center. The resulting restrictions on campus attendance and patient contact have triggered the adjustment of our ear examination course for undergraduate medical students. Here, we report the insights developed from this experience and the implications for the undergraduate teaching of ear examination in the post COVID-19 era, especially with regard to the examination tools and techniques.

Ear examination has undergone many changes throughout history from tongs, ear specula and head mirrors to handheld otoscopes and, in recent decades, binocular microscopes and endoscopes [2]. Notably, the handheld otoscope has been the standard tool of ear examination since the late nineteenth century. Aside from otolaryngologists and otologists, who may additionally use binocular microscopes and endoscopes for ear examination, all other medical professionals, such as pediatricians, family physicians and emergency physicians, solely rely on handheld otoscopes for ear examination [3], [4], [5], [6]. With the advent of endoscopic ear surgery [7], [8], [9], an increasing number of otolaryngologists have started to use endoscopes for office examinations [10], [11] due to the method’s superior illumination, excellent image quality and possibility of photo/video documentation [12], [13]. Most importantly, the endoscope is the only examination tool that allows the examiner to both obtain a wide panoramic view and physically approximate the tympanic membrane for close inspection [12], [13].

Undergraduate medical students usually do not receive exposure to endoscopic ear examination in their teaching programs. Previous studies have shown that medical students are not sufficiently confident in their diagnostic ear examination skills, suggesting that the undergraduate teaching of ear examination is inadequate [5], [14], [15]. Even postgraduate trainees and physicians have reported insufficient comfort levels with otoscopy [16], [17]. Nevertheless, handheld otoscopy remains the standard ear examination tool used in undergraduate medical studies, despite the associated suboptimal student performance levels.

The aim of this study is to assess our preliminary experience with teaching endoscopic ear examination to undergraduate medical students and to survey the students with regard to their confidence level with this examination technique and the method’s overall preference compared with that of handheld otoscopy. This pilot undergraduate teaching program was designed during the COVID-19 pandemic, aiming to maximize the diagnostic skills of medical students while minimizing the time spent on campus attendance and patient contact. The present study examined the research hypothesis whether the introduction of ear endoscopy to undergraduate medical students improved their self-reported confidence levels during ear examination and whether the students prefer this added technique and recommend it for future medical students. The theoretical framework of the study is based on the increasing literature about the benefits of endoscopy in ear examination and surgery in the field of otolaryngology, while still being completely unfamiliar to undergraduate learners and non-otolaryngologic postgraduate learners [10], [11], [12], [13].

## 2. Project description

**Figure 1:**
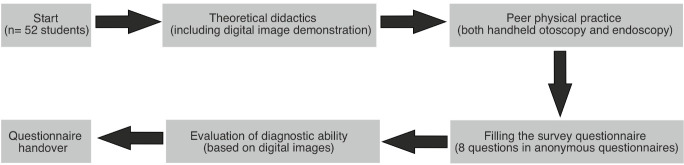
Schematic illustration of the course workflow

**Figure 2:**
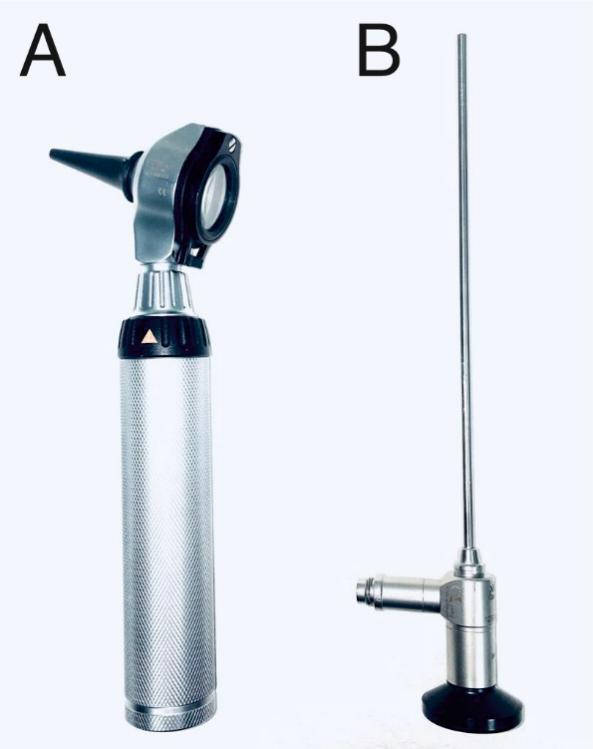
Photograph showing a handheld otoscope (A) and 0-degree 4-mm rigid endoscope (B).

The study involved the survey of undergraduate medical students with little to no previous experience in ear examination. The students underwent a two-hour-long teaching program based on basic ear examination that consisted of theoretical didactics, exemplary digital endoscopic images and peer physical practice (PPE). A schematic illustration of the course workflow is illustrated in figure 1 (Fig. 1). Peer physical practice was performed with both a handheld otoscope (Beta100; Heine Optotechnik, Gilching, Germany) and a 0-degree rigid endoscope (125 304 120; XION GmbH, Berlin, Germany) attached to an LED light source (11301 D4; Karl Storz Endoskope, Tuttlingen, Germany) (see figure 2 (Fig. 2)). After endoscopic examination of each subject, the endoscope was disinfected using the Tristel^®^ Trio Wipes System (Tristel GmbH, Berlin, Germany). All the students were taught and practiced both techniques of ear examination, with one student simulating the role of the patient and the other students simulating the role of the examiner. All students tested negative for SARS-CoV-2 directly before their course attendance. The students all wore nose-mouth face masks for the entire duration of the course, and social distancing was practiced as much as possible while still allowing for peer practice.

Afterwards, the students were asked to fill out a survey questionnaire consisting of eight questions. The first two questions pertained to the students’ gender and previous experience with ear examination. The subsequent six questions were measured on a 5-point Likert scale, and evaluated the reactionary level of the learning experience according to the Kirkpatrick model [18]. The survey questions are shown in table 1 (Tab. 1). To evaluate their diagnostic performance (results level of the Kirkpatrick model [18]), the students were asked to view colored digital endoscopic images on a computer screen and to identify common middle ear pathologies, which were introduced during the theoretical part of the course (10 exemplary digital images viewed on a computer screen for each pathology). The following four middle ear pathologies were included in this brief assessment: acute otitis media, tympanic membrane perforation, otitis media with effusion, and tympanic membrane retraction. The students were asked to choose the correct underlying pathology in a paper questionnaire containing multiple choice questions. The second and third levels of the Kirkpatrick model (learning level and behavior level, respectively) were not evaluated in this study. Statistical analysis was performed using JMP software version 15 (SAS Institute, Cary, NC, USA).

## 3. Results

**Figure 3:**
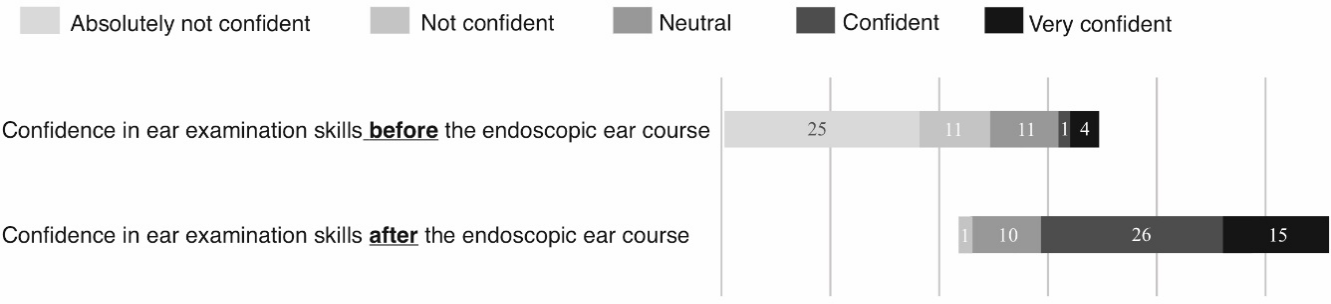
Bar graphic representation of the results of questions (c) and (d) of the survey questionnaire (n=52 students for all questions). The results were reported on the following 5-point Likert scale: (1): absolutely not confident (definitely not able); (2): not confident (probably not able); (3): neutral (possibly able); (4): confident (likely able); (5): very confident (probably able).

The survey questionnaire and the performance evaluation were completed by fifty-two undergraduate medical students (32 females and 20 males) in either the second or fourth year of their medical studies. The survey started with one demographic question (gender) and one question about the respondent’s previous experience in ear examination. All the students (n=52) reported having little to no previous experience with ear examination (less than 5 times). The second-year medical students were complete novices in regard to ear examination (n=40), while the fourth-year medical students (n=12) had received a little exposure to ear examination in the course of their studies (at least once but less than 5 times). The overall self-reported confidence level regarding ear examination before the teaching program was generally low, averaging a score of 2 on a 5-point Likert scale (see figure 3 (Fig. 3)). The self-reported confidence levels before the course were related to the students’ previous experience with ear examination (p<0.05, Pearson’s Chi square test). The students’ overall self-reported confidence levels regarding ear examination increased after the two-hour practical course to an average of 4 (on a scale of 1 to 5), suggesting that the students could reach a sufficient level of confidence in their diagnostic ability after a two-hour practical course consisting of a theoretical introduction and peer practice (see figure 3 (Fig. 3)). There was no difference between the second-year and fourth-year students with regard to the self-reported confidence levels after the course (p>0.05, Student’s t-test).

**Figure 4:**
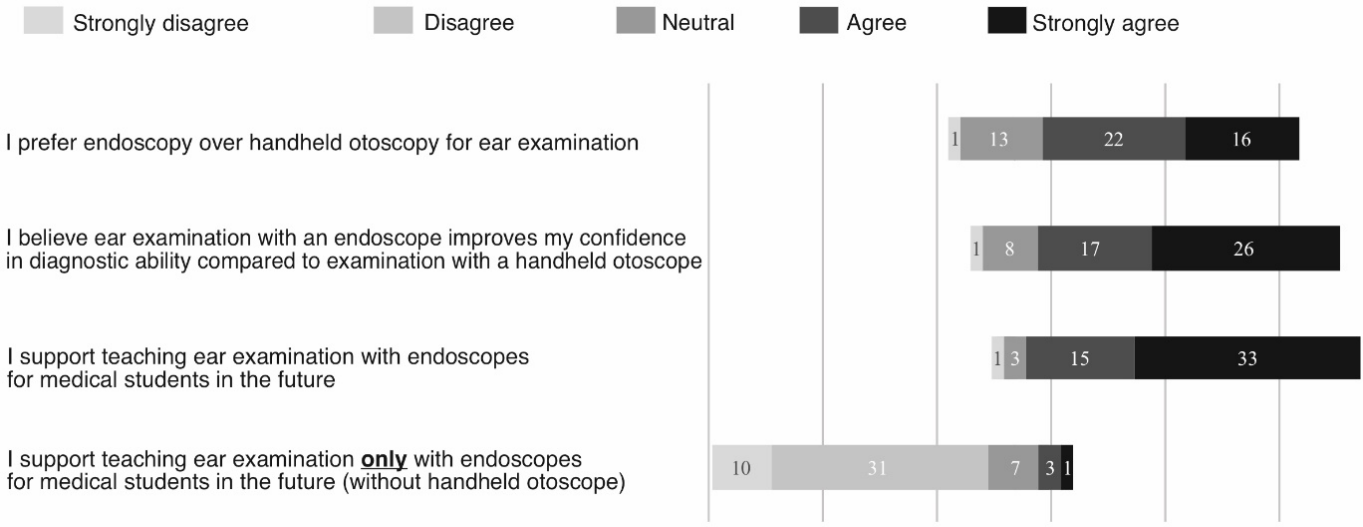
Bar graphic representation of the results of the questions (e, f, g, h) of the survey questionnaire (n=52 students for all questions). The results were reported on the following 5-point Likert scale: (1): strongly disagree; (2): disagree; (3): neutral; (4): agree; (5): strongly agree.

The next four questions in the survey compared the students’ opinions of handheld otoscopy and endoscopy as ear examination tools (see figure 4 (Fig. 4)), which represents the reactionary level of the learning experience, according to the Kirkpatrick model [18]. Most students expressed either a preference (n=22, 42.3%) or a strong preference (n=16, 30.7%) for ear examination with an endoscope rather than that with a handheld otoscope (average 4 out of 5). Most students either agreed (n=17, 32.6%) or strongly agreed (n=26, 50%) that their confidence in their diagnostic ability with an endoscope was higher than that with a handheld otoscope (average 4.2 out of 5).

The final two questions surveyed the students’ recommendations for the future teaching of ear examination to medical students. Most students either supported (n=15, 28.8%) or strongly supported (n=31, 59.6%) the teaching of ear examination with an endoscope to future medical students as an adjunct to handheld otoscopy (average of 4.5 out of 5). Only one student (3.2%) recommended against teaching endoscopic ear examination in future teaching programs. In contrast, the medical students were significantly less enthusiastic about teaching ear examination exclusively with an endoscope as a substitute for handheld otoscopy (Student’s t-test, p<0.001), with most students either disagreeing (n=31, 59.6%) or strongly disagreeing (n=10, 19.2%) with this suggestion, and 7 (13.4%) students remaining neutral (average agreement of 2.1 out of 5). These results suggest that inexperienced medical students support the teaching of endoscopic ear examination in future undergraduate programs in conjunction with handheld otoscopy.

**Figure 5:**
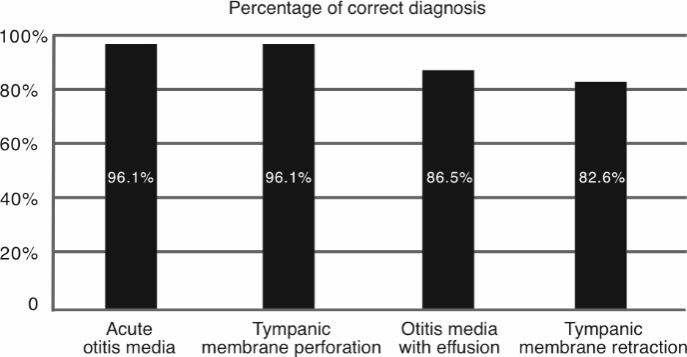
The number and percentage of students who correctly identified the correct diagnosis in a series of digital endoscopic images of the following four common middle ear pathologies: acute otitis media, tympanic membrane perforation, otitis media with effusion, and tympanic membrane retraction (52 students overall).

With regard to the “Results” level of learning (according to the Kirkpatrick model [18]), the diagnostic ability of the students was assessed based on digital endoscopic images. The students were asked to identify the correct underlying diagnosis in a series of exemplary images representing four common middle ear pathologies (see figure 5 (Fig. 5)). Out of all 52 students, 50 (96.1%) could correctly identify acute otitis media on digital endoscopic images. Similarly, 50 out of 52 students (96.1%) could correctly identify a tympanic membrane perforation. In contrast, students were less likely to identify otitis media with effusion and tympanic membrane retraction, with 45 (86.5%) and 43 (82.6%) students correctly identifying these pathologies, respectively. These findings suggest that the diagnosis of middle ear effusion and tympanic membrane retraction are more difficult to teach to inexperienced medical learners based solely on digital exemplary images and thus require more extensive practice and the examination of real patients with those pathologies. There were no significant differences in the percentage of correct answers between younger and older students, or between student self-reported confidence scores and their accuracy in determining the correct diagnosis in exemplary digital images. Notably, this digital image demonstration and evaluation was performed for all students at the end of the course, and was not assessed differentially in relation to the examination method or the self-reported student confidence, but rather was used as a general indicator of the applicability of this teaching platform for providing adequate understanding of normal and pathologic ear findings. Another aim of the digital image section was to provide insight into the differential level of diagnostic difficulty of various common ear pathologies for medical students, in order to guide future evaluations of the results following the Kirkpatrick model [18]. Further studies should focus on the comparison of diagnostic accuracy between student groups trained in either examination method separately.

## 4. Discussion

In this study, the students performed well in their diagnostic evaluations after being introduced to ear endoscopy and digital endoscopic images of various ear pathologies. The students expressed an overall preference for ear examination with an endoscope compared to that with a handheld otoscope, which may suggest that handheld otoscopy is more difficult for inexperienced learners who have no previous experience with ear examination. This finding mirrors our experience in the postgraduate teaching of our otolaryngology residents, who increasingly tend to prefer ear endoscopy over the traditional otoscopic examination in recent years. It is important to note that the learning objectives in official undergraduate curricula of all German-speaking countries currently do not include endoscopic ear examination. This lack of endoscopic ear teaching in official curricula was the main motivation for this study.

One main goal of medical education is to fulfil the educational needs of the student. We conclude that students should be trained in their preferred examination methods to achieve the highest levels of confidence and skill, especially in the age of self-regulated learning and student-centered medical education [19]. We thus propose taking into consideration the students’ own support for teaching endoscopic ear examination in the future. Another strategy to produce better teaching outcomes is the standardization of the examination process, which leads to improved objective quality of the teaching and examination processes, as shown by previous studies [20], [21]. 

Interestingly, most students did not support the teaching of ear examination solely with endoscopy, suggesting that endoscopy should be an addition to, and not a substitute for, handheld otoscopy in undergraduate teaching programs. The restrictions on patient contact and examination, which have been imposed due to the COVID-19 pandemic, prevented a direct comparison of the diagnostic performance of the students using both methods on patients with real ear pathologies. Instead, we focused on surveying the students regarding their overall preference and self-reported confidence in their examination skills with both methods in peer practice. Future studies should directly evaluate the diagnostic performance of medical students using both examination methods.

In this survey, most medical students supported the teaching of ear examination using an endoscope as an adjunct tool, but not as an alternative to the handheld otoscope, which remains the standard examination tool among non-otolaryngologic clinicians. This recommendation fits to our view of the endoscope as an adjunct tool, rather than a replacement for the handheld otoscope. In addition, the necessary time and cost of endoscope disinfection between examinations represents a disadvantage compared to handheld otoscopy, which employs disposable specula and is thus more applicable to real-life patient examinations in busy practices. Indeed, postgraduate non-otolaryngologic medical practitioners will likely only have access to handheld otoscopes in their postgraduate training programs and practices as the sole ear examination tool. Thus, it appears appropriate that the undergraduate medical students are trained in the examination technique that they are most likely to practice in their postgraduate careers. We therefore propose that the undergraduate teaching of ear examination should be performed using both a handheld otoscope in addition to an endoscope. The additional use of the endoscope as an adjunct teaching tool improves the subjective diagnostic confidence of medical learners without depriving them of the exposure to handheld otoscopy, which they are more likely to practice during their postgraduate training. 

We believe that a higher self-reported confidence level should be one of the main goals of any skill teaching module, although such outcomes do not always correlate to better diagnostic performance [22]. Thus, we propose that undergraduate teaching programs should invest in endoscopy and digital camera systems for the teaching of ear examination. The potential to attach digital cameras to endoscopes allows the teacher (and other students) to simultaneously observe what the examiner is seeing, and vice versa, thereby resulting in a more controlled and uniform learning experience for all students regardless of their individual performance skills. In addition, camera recordings are conducive to both virtual learning and the exchange of experience among students and teachers.

It is important to note that this teaching module is not an alternative to the examination of patients with real pathologies [23], which has not been possible during the COVID-19 pandemic. However, even without patient contact, we demonstrated that certain common ear pathologies are more amenable to digital learning, such as acute otitis media. This finding is of interest since acute otitis media is commonly encountered by general practitioners, pediatricians and emergency physicians. In contrast, other ear pathologies, such as tympanic membrane retraction, are subtler and may require more practice on real patients. One method to allow practice without patient contact would be to use otoscopy simulators, as reported previously [22]. In future studies, it would be interesting to compare the diagnostic performance of novice medical learners on patients with subtle ear pathologies using both handheld otoscopy and endoscopy to determine if the use of endoscopes indeed results in better diagnostic skills.

To our knowledge, this is the first report of teaching rigid ear endoscopy to undergraduate medical students and surveying their reaction to this examination method in comparison to the conventional handheld otoscopy. While not offering an educational intervention that directly compares the merits of both examination methods, we argue this study may still have important implications for the teaching of ear examination in undergraduate, and even postgraduate, programs. The present survey study clearly demonstrates the strong preference for ear endoscopy over handheld otoscopy among a cohort of undergraduate medical students. Thus, handheld otoscopy appears to be more difficult to master and therefore more suitable for advanced learners. This higher difficulty level of handheld otoscopy may contribute to the poor otoscopy skills among medical students that have been reported in the literature. Thus, the early exposure of novice medical learners to endoscopic ear examination may help reduce this problem. The results of the present pilot study will influence the future teaching of ear examination in our undergraduate otolaryngology program in the post-COVID-19 era. Future interventional studies should compare the diagnostic performance of the medical students with both examination methods, which would allow to issue clear recommendations on the role of endoscopy in the undergraduate teaching curriculum of ear examination.

## Acknowledgement

The authors thank the medical students who participated in the survey.

## Authorship

The authors Philipp Arens and Katharina Stölzel share the last authorship.

## Competing interests

The authors declare that they have no competing interests. 

## Figures and Tables

**Table 1:**
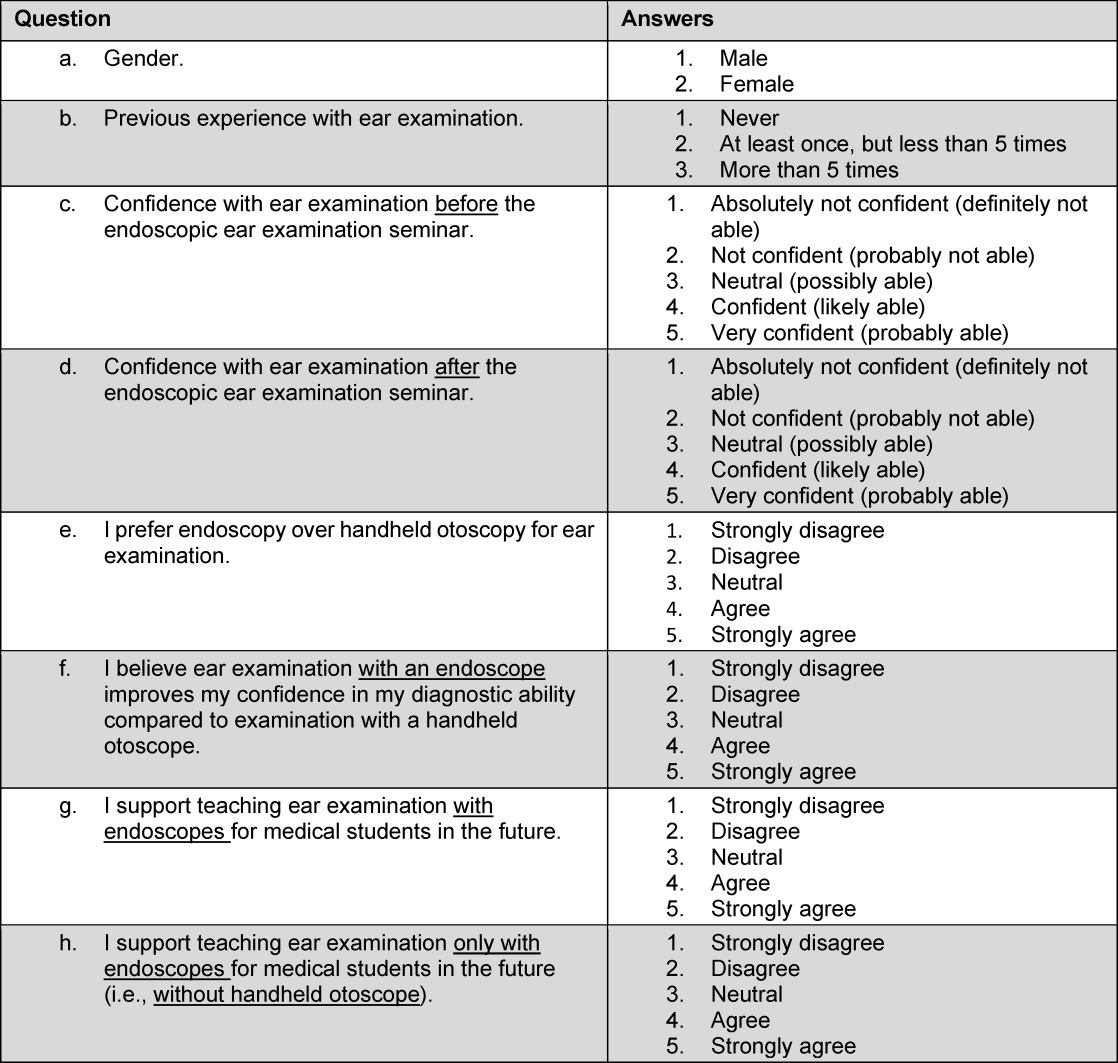
The questionnaire used in the survey
